# Framing chronic absenteeism and emotionally-based school absenteeism as public health problems

**DOI:** 10.3389/frcha.2025.1662093

**Published:** 2025-08-21

**Authors:** Christopher A. Kearney

**Affiliations:** Department of Psychology, University of Nevada, Las Vegas, Las Vegas, NV, United States

**Keywords:** chronic school absenteeism, emotionally-based school absenteeism, public health, ecological, systems and policy, epidemiologic and statistical, environmental and occupational, behavioral and social science

## Abstract

Chronic school absenteeism (CSA) and emotionally-based school absenteeism or avoidance (EBSA) are highly prevalent conditions linked to multiple short- and long-term problems across academic, social-emotional, physical and mental health, family, and occupational and economic domains of functioning. In addition, CSA and EBSA occur disproportionately across vulnerable student groups and have been the focus of extensive preventative and intervention efforts. As such, CSA and EBSA may meet criteria as formal public health problems. This perspective article illustrates various ways of framing CSA and EBSA in this fashion utilizing contemporary public health models. Categories of public health models are emphasized in this regard and include ecological, systems and policy, epidemiologic and statistical, environmental and occupational, and behavioral and social science approaches. Each approach closely parallels research and other work regarding school absenteeism. The article is designed as a step toward advocacy for recognizing CSA and EBSA as formal public health problems contingent upon consensus among key constituencies in this area.

## 1 Introduction

Chronic school absenteeism (CSA) and emotionally-based school absenteeism (or avoidance) (EBSA) have become increasingly severe problems among children and adolescents. The prevalence of CSA, often defined as missing at least 10% of school days, has risen in recent years among European [e.g., 20% in England (1)]; and American (28%) youth (2). Worldwide, 16% of children are not in school at all, a rate that is particularly elevated in central and southern Asia and parts of Africa (3). The prevalence of EBSA, defined here as school absences impacted by anxiety and mood disorders and related emotional conditions, sometimes also referred to as school refusal, is less well-documented and may be as high as 35% (4). Rates of emotional disorders among youth have skyrocketed in recent years and often co-occur with school absenteeism (5, 6). In addition, extensive school absenteeism is associated with significant impairment in academic, social-emotional, mental and physical health, and family domains of functioning (7). School absenteeism and school dropout are also associated with substantial, adverse long-term outcomes across economic, occupational, and health-related domains (8).

CSA and EBSA are addressed by professionals across a wide swath of disciplines such as child development, criminal and juvenile justice, education, medicine, psychology, public policy, and social work, among many others. This has led to a rich literature base and several common core assumptions: (a) school attendance/absenteeism are global issues; (b) school attendance/absenteeism rates differ substantially and especially across vulnerable student groups; (c) school attendance is generally associated with positive student outcomes and school absenteeism is generally associated with negative student outcomes; (d) school attendance/absenteeism are complex constructs impacted by multiple risk and protective factors; (e) positive interventions to enhance school attendance and reduce school absenteeism are generally but moderately effective (9). At the same time, however, disparate perspectives in this area have produced a scattered and uncoordinated set of terminologies, classification strategies, and assessment and intervention practices for CSA and EBSA (10). Calls have thus been made for integrative frameworks to enhance synthesis and collective action regarding these complex constructs [e.g., (11)].

One avenue for integrating various perspectives in this area is to formally recognize or declare CSA and EBSA to be *public health problems*. A public health problem is typically defined as an issue that negatively affects the health of a population, in this case primarily children and adolescents. Although often applied to medical conditions, public health problems have also encompassed nonmedical conditions such as social disconnection or intimate partner or community violence (12, 13). A public health problem may be differentiated from a public health *crisis*, the latter of which usually involves a particularly urgent situation that requires immediate, focused, and often emergency action to mitigate significant harm to a large population. Although CSA and EBSA are harmful, the conditions may not currently rise to the level of a public health crisis in most areas.

Framing an issue as a public health problem promotes more coordinated and comprehensive approaches to address the problem and carries several advantages. First, identification as a public health problem stimulates the mobilization of resources such as funding, personnel, and time to address the problem effectively. Second, identification as a public health problem advances the development and implementation of interventions that can most benefit the entire population, in addition to specific groups. Third, identification as a public health problem spurs policy development and advocacy in the direction of practices that best promote student health and well-being. Fourth, identification as a public health problem facilitates prevention and health promotion strategies to mitigate severe problems. Fifth, identification as a public health problem emphasizes a population-based approach and spotlights important health disparities among different demographic groups. Finally, identification as a public health problem promotes increased awareness and public knowledge of the problem at hand [e.g., (14, 15)].

CSA and EBSA match general criteria for identifying a particular problem as public health-oriented. These criteria include burden of disease/condition, vulnerable populations, and availability of solutions (16). Burden of disease/condition refers to substantial and negative overall impact on a population, including social, economic, and other costs. Vulnerable populations refer to disproportional impact on specific groups (health disparities) vis-à-vis demographic, geographic, or income and other variables. Availability of solutions refers to the presence of effective preventative and treatment options that could be applied to the public health problem. These conditions apply well to CSA and EBSA (17, 18).

## 2 Contemporary public health models

**Figure 1:**
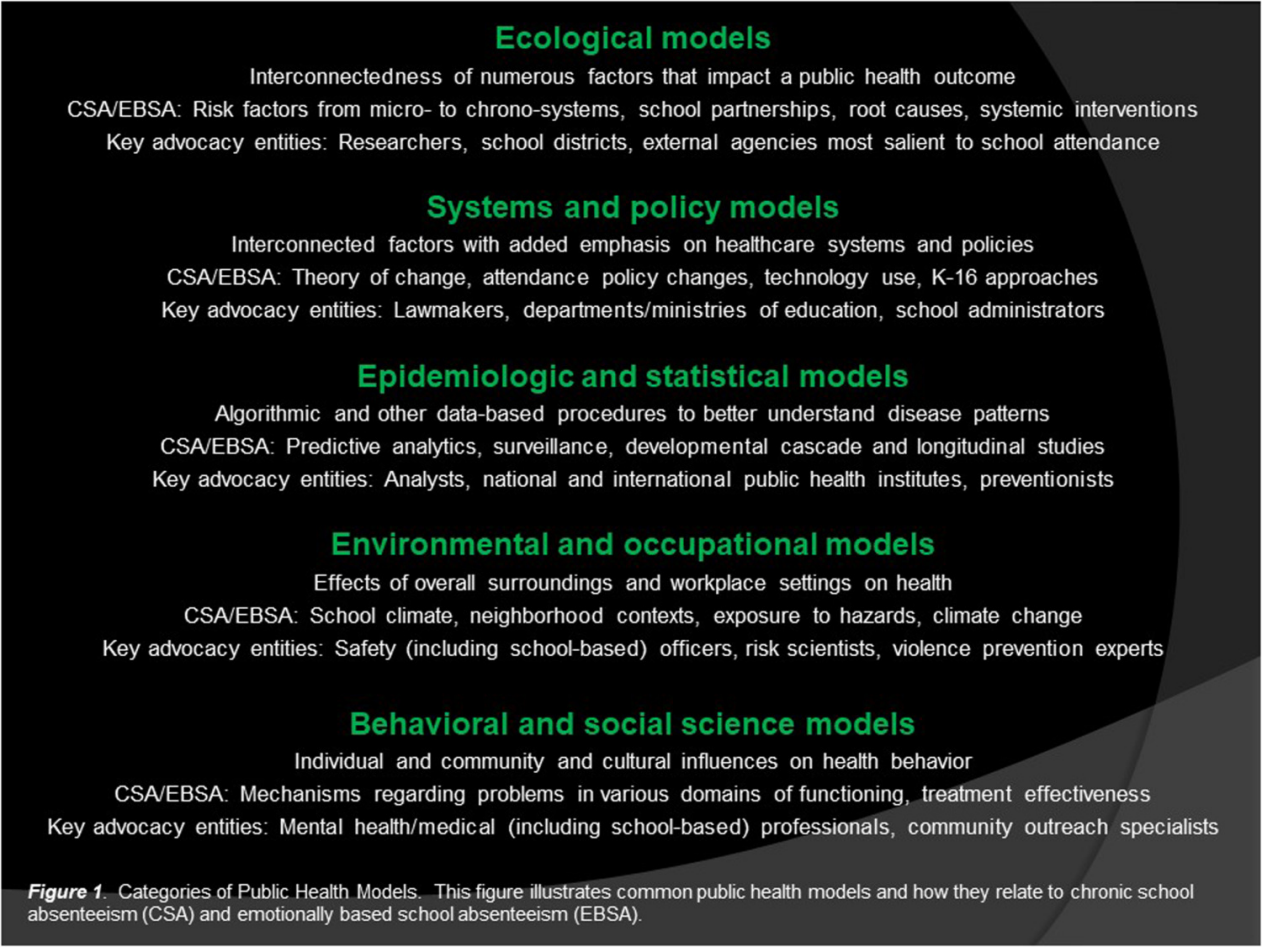
Categories of public health models. This figure illustrates common public health models and how they relate to chronic school absenteeism (CSA) and emotionally based school absenteeism (EBSA).

The purpose of this perspective article is to illustrate various ways of framing CSA and EBSA as formal public health problems utilizing contemporary public health models. The following sections outline common categories of public health models (i.e., ecological, systems and policy, epidemiologic and statistical, environmental and occupational, behavioral and social science) and demonstrate how each parallels research and other work regarding school absenteeism (Figure 1). The article is designed as a step toward advocacy in this area should stakeholders actively contend that school absenteeism is indeed a population-level public health problem.

### 2.1 Ecological models

Ecological models of public health emphasize the interconnectedness of numerous factors that impact a particular health outcome. These factors are typically organized into levels of influence to recognize the fact that individuals interact with their surrounding environment and that health conditions are thus influenced by multiple levels of determinants. Many of these determinants are nonmedical in nature and can include living environments, access to quality education and healthcare, financial stability, and social contexts, among others (19). Ecological models of public health have several variations (e.g., socio-ecological model) but many center on individual, interpersonal, organizational, community, and other variables to understand how complex health outcomes are shaped (20). Ecological models are also used to develop comprehensive health interventions by addressing factors at each level.

Ecological models of public health are quite amenable to CSA and EBSA. Researchers and other stakeholders in this area often gravitate to ecological approaches that emphasize multiple levels of risk and protective factors (21). These levels parallel classical ecological systems theory (22). Key risk factors for CSA/EBSA, for example, are commonly arranged across microsystem (e.g., child anxiety), mesosystem (e.g., child-parent/peer conflict), exosystem (e.g., school support), macrosystem (e.g., transportation vulnerability), and chronosystem (e.g., primary vs. secondary school) levels (23). In addition, treatments for CSA and EBSA are designed to enhance family-school-practitioner partnerships to address multiple contextual variables at various levels (24). Others utilize ecological approaches to understand root causes and specific systemic interventions necessary to impact widespread CSA in given districts and geographical areas [see, for example, (25)]. Key entities for advocacy vis-à-vis ecological approaches include researchers, school districts, and external agencies particularly salient to school attendance (e.g., transportation).

### 2.2 Systems and policy models

Systems and policy models of public health also emphasize the importance of various interconnected factors for shaping health conditions, with an added and specific emphasis on healthcare systems and policies. As such, systems and policy models of public health are often utilized for large-scale, structural, or policy-level interventions. Examples include increased taxes on certain products, improved access to nutritious foods, zoning regulations, and harm reduction practices. Systems and policy models of public health have several variations (e.g., PRECEDE–PROCEED model) that emphasize a collective stakeholder approach to identify key problems and needs (and desired outcomes) and implement and evaluate solutions (26). Such models often work in backwards fashion to identify positive long-term outcomes, conditions that lead to such outcomes, and earliest changes that must occur in the current environment (27).

Systems and policy models of public health are quite amenable to CSA and EBSA. A theory of change approach, for example, has been proposed for these problems (9). This approach centers on desired long-term goals or outcomes (e.g., readiness for adulthood), intermediate steps or outputs that produce such outcomes (e.g., enhancing attendance, social justice, shared alliances), and current conditions or inputs that might propel the outputs (e.g., changes in education, technology, climate). A theory of change creates a mutual vision among disparate stakeholders and can facilitate large-scale, structural, or policy-level interventions. Examples include eliminating inequities in how students are penalized for absenteeism (including exclusionary discipline) and enacting practices that foster tiered systems of support to boost attendance (28). Other applied examples might include adapting technology to better reach absent students and developing K-16 educational approaches to enhance flexible readiness pathways (29). Key entities for advocacy vis-à-vis systems and policy approaches include lawmakers, departments/ministries of education, and school administrators.

### 2.3 Epidemiologic and statistical models

Epidemiologic and statistical models of public health emphasize algorithmic and other data-based procedures to better understand disease trends and patterns, risk factors, and effective public health strategies. These data are also used for predictive purposes to forecast future areas of concern and thus assign resources more proactively to enhance public health (30). Examples include issuing public warnings, closing schools, and developing more effective vaccines. Epidemiologic and statistical models of public health have several variations (e.g., triad, web of causation, disease transmission) that commonly emphasize a relationship between an agent, host, and environment to study how a condition develops and becomes distributed and to inform potential interventions [e.g., (31)]. In many such models, cases are categorized according to those susceptible, infected, and recovered and how people may move between these groups. Such models may be particularly useful for understanding chronic and complex public health problems that involve an interplay of multiple elements.

Epidemiologic and statistical models of public health are quite amenable to CSA and EBSA. The advent of large data sets in education and other (e.g., housing) agencies allows for machine learning, artificial intelligence, and predictive analytics to examine systemic root causes of school absenteeism in a given area and to track students separated from the educational process. Large data algorithmic and other analysis has been used, for example, to identify patterns of residential mobility and household and other variables that drive school absenteeism (32). School absenteeism is also used as a surveillance method for disease (33). Epidemiologic and statistical models that focus on how a condition develops and becomes distributed are also amenable to CSA and EBSA. Developmental cascade models of school absenteeism, for example, focus on how risk factors emerge and metastasize over time. An example is progression from school disengagement to school absenteeism to school dropout (34). Such models may be useful for clinical applications as well. Longitudinal work, for example, illustrates that children with emotional difficulties are at greater risk for specific types of school absences, which may help refine screening and early intervention efforts (6). Key entities for advocacy vis-à-vis epidemiologic and statistical approaches include analysts, national and international public health institutes, and preventionists.

### 2.4 Environmental and occupational models

Environmental and occupational models of public health emphasize how overall surroundings and workplace settings impact individual and population health. A focus is often made on understanding exposure to specific hazards and their dosage levels to control and minimize negative health impacts and to develop effective prevention practices (35). Examples include limiting exposure to chemicals, implementing safety regulations, and monitoring air and water quality. Environmental and occupational models of public health have several variations (e.g., risk assessment, environmental health paradigm) that commonly emphasize a relationship between hazard sources, exposure, dose, effect, and response (36). Such models may be especially useful for protecting particularly vulnerable populations, reducing instances of injury, and mitigating long-term effects of hazardous exposure.

Environmental and occupational models of public health are quite amenable to CSA and EBSA. Researchers in this area often focus, for example, on school climate and neighborhood contexts with respect to school absenteeism. Key dimensions of school climate commonly associated with school absenteeism include degree of connectedness with peers and teachers, engagement in school activities, and safety (37). In addition, neighborhood-level variables such as resource deprivation, violence, and dangerous avenues to school impact absenteeism (38). Specific dosage levels are sometimes a part of research in this area as well, such as the number of adverse childhood experiences associated with levels of school absenteeism (39). Surrounding environmental conditions also relate to school absenteeism, including exposure to industrial hazards and pollution as well as dilapidated or inadequate physical facilities at school (40). Effects of climate change impact school absenteeism at proximal and distal levels as well (41). Each of these avenues of study provide potential targets for early and later intervention. Key entities for advocacy vis-à-vis environmental and occupational approaches include safety (including school-based) officers, risk scientists, and violence prevention experts.

### 2.5 Behavioral and social science models

Behavioral and social science models of public health emphasize individual and community and cultural influences on health behavior. A focus is often made on nonmedical factors that promote and deter healthy and harmful practices and inform interdisciplinary or biopsychosocial interventions (42). Examples include understanding factors that motivate early screening, healthy lifestyle habits, and compliance to treatment regimens and factors that reduce risky behaviors and stigma and other barriers to care. Behavioral and social science models of public health have several variations (e.g., health belief, theory of planned behavior, transtheoretical, social cognitive theory) that commonly emphasize a relationship between various psychological factors and health-based actions (43). Such factors can involve self-efficacy, decision-making, intentions, attitudes, and perceptions of health-based barriers, benefits, control, severity, susceptibility, and threats, among other beliefs. Such factors can intersect with broader processes such as social support, observational learning, and cultural contexts in dynamic or reciprocal fashion to influence health behaviors. Behavioral and social science models of public health incorporate these interconnected factors to better understand resistance to behavior change, consideration and action regarding health-based behaviors, and sustained behavior change toward positive health outcomes (44). Such insights can also inform effective public health interventions targeted to these various stages.

Behavioral and social science models of public health are quite amenable to CSA and EBSA. Researchers often focus on specific mechanisms that explain the relationship between school absenteeism and problems in various domains of functioning. Examples include mechanisms for how school absenteeism relates to problems in academic (e.g., less teacher-based instruction), social-emotional (e.g., fewer friendships), mental health (e.g., stress), physical health (e.g., medical appointments), and family (e.g., conflict) functioning (7). In addition, a focus is often made on mechanisms of mental health and other treatment that best support a return to school attendance. Examples include improvements in self-efficacy, sleep, coping ability, and social and academic competence [e.g., (45)]. Indeed, a lengthy clinical research history is associated with various forms of CSA and EBSA. Key entities for advocacy vis-à-vis behavioral and social science approaches include mental health and medical (including school-based) professionals and community outreach specialists.

## 3 Conclusion

Chronic school absenteeism and emotionally-based school absenteeism/avoidance could be considered critical public health problems, but only if those from various constituencies actively advocate for such an approach, and in conjunction with student and family voices. Advocacy in this regard involves gathering evidence from different perspectives, building coalitions, and communicating the scope and impact of CSA and EBSA to relevant stakeholders to drive change. Fortunately, CSA and EBSA have been examined via multiple approaches that parallel contemporary public health models. As such, the approaches may resonate well with professionals who work to protect and improve population health via prevention and promotion of well-being. School attendance and education provide the foundation for a healthy and fulfilling life. Adopting a public health approach to augment these processes would thus seem natural and morally appropriate.

## Data Availability

The original contributions presented in the study are included in the article/Supplementary Material, further inquiries can be directed to the corresponding author.

## References

[1] UK Department of Education. (2025). Academic year 2023/24: Pupil absence in schools in England. Available online at: https://explore-education-statistics.service.gov.uk/find-statistics/pupil-absence-in-schools-in-england/2023-24

[2] Malkus N. Long COVID for Public Schools: Chronic Absenteeism Before and After the Pandemic. Washington, DC: American Enterprise Institute (2024).

[3] UNESCO. 250 Million Children out-of-School: What You Need to Know About UNESCO’s Latest Education Data. Paris: Author (2023).

[4] Fernandes CSF Kannoth S Pendergrass Boomer TM Hieftje KD Fiellin LE. Systematic review of interventions with some school involvement for school refusal in high school-age adolescents. Child Sch. (2024) 46:85–95. 10.1093/cs/cdae003

[5] Elia J Pajer K Prasad R Pumariega A Maltenfort M Utidjian L Electronic health records identify timely trends in childhood mental health conditions. Child Adolesc Psychiatry Ment Health. (2023) 17:107. 10.1186/s13034-023-00650-7 37710303 PMC10503059

[6] Panayiotou M Finning K Hennessey A Ford T Humphrey N. Longitudinal pathways between emotional difficulties and school absenteeism in middle childhood: evidence from developmental cascades. Dev Psychopathol. (2023) 35:1323–34. 10.1017/S095457942100122X 34955109

[7] Kearney CA Dupont R Fensken M Gonzálvez C. School attendance problems and absenteeism as early warning signals: review and implications for health-based protocols and school-based practices. Front Educ. (2023) 8:1253595. 10.3389/feduc.2023.1253595

[8] Rocque M Jennings WG Piquero AR Ozkan T Farrington DP. The importance of school attendance: findings from the Cambridge study in delinquent development on the life-course effects of truancy. Crime Delinq. (2017) 63:592–612. 10.1177/0011128716660520

[9] Kearney CA Benoit L Gonzálvez C Keppens G. School attendance and school absenteeism: a primer for the past, present, and theory of change for the future. Front Educ. (2022) 7:1044608. 10.3389/feduc.2022.1044608

[10] Havik T Ingul JM. How to understand school refusal. Front Educ. (2021) 6:715177. 10.3389/feduc.2021.715177

[11] Allensworth E Balfanz R Rogers T Demarzi J. Absent from School: Understanding and Addressing Student Absenteeism. Cambridge, MA: Harvard Education Press (2021).

[12] Holt-Lunstad J. Social connection as a public health issue: the evidence and a systemic framework for prioritizing the “social” in social determinants of health. Annu Rev Public Health. (2022) 43:193–213. 10.1146/annurev-publhealth-052020-110732 35021021

[13] Stöckl H Sorenson SB. Violence against women as a global public health issue. Annu Rev Public Health. (2024) 45:277–94. 10.1146/annurev-publhealth-060722-025138 38842174

[14] Brady SS Brubaker L Fok CS Gahagan S Lewis CE Lewis J Development of conceptual models to guide public health research, practice, and policy: synthesizing traditional and contemporary paradigms. Health Promot Pract. (2020) 21:510–24. 10.1177/1524839919890869 31910039 PMC7869957

[15] Dykxhoorn J Fischer L Bayliss B Brayne C Crosby L Galvin B Conceptualising public mental health: development of a conceptual framework for public mental health. BMC Public Health. (2022) 22:1407. 10.1186/s12889-022-13775-9 35870910 PMC9308351

[16] Jit M Cook AR. Informing public health policies with models for disease burden, impact evaluation, and economic evaluation. Annu Rev Public Health. (2023) 45:133–50. 10.1146/annurev-publhealth-060222-025149 37871140

[17] Eklund K Burns MK Oyen K DeMarchena S McCollom EM. Addressing chronic absenteeism in schools: a meta-analysis of evidence-based interventions. School Psych Rev. (2022) 51:95–111. 10.1080/2372966X.2020.1789436

[18] Maynard BR Heyne D Brendel KE Bulanda JJ Thompson AM Pigott TD. Treatment for school refusal among children and adolescents: a systematic review and meta-analysis. Res Soc Work Pract. (2018) 28:56–67. 10.1177/1049731515598619

[19] Office of Disease Prevention and Health Promotion. Social Determinants of Health. Healthy People 2030. Washington, DC: U.S. Department of Health and Human Services (n.d.). Available online at: https://health.gov/healthypeople/objectives-and-data/social-determinants-health

[20] Caperon L Saville F Ahern S. Developing a socio-ecological model for community engagement in a health programme in an underserved urban area. PLoS One. (2022) 17:e0275092. 10.1371/journal.pone.0275092 36155664 PMC9512167

[21] Melvin GA Heyne D Gray KM Hastings RP Totsika V Tonge BJ The kids and teens at school (KiTeS) framework: an inclusive bioecological systems approach to understanding school absenteeism and school attendance problems. Front Educ. (2019) 4:1–9. 10.3389/feduc.2019.00061

[22] Bronfenbrenner U. Developmental ecology through space and time: a future perspective. In: Moen P Elder GH Jr. Lüscher K, editors. Examining Lives in Context: Perspectives on the Ecology of Human Development. Washington, DC: American Psychological Association (1995). p. 619–47. 10.1037/10176-018

[23] Gubbels J van der Put CE Assink M. Risk factors for school absenteeism and dropout: a meta-analytic review. J Youth Adolesc. (2019) 48:1637–67. 10.1007/s10964-019-01072-5 31312979 PMC6732159

[24] Leduc K Tougas AM Robert V Boulanger C. School refusal in youth: a systematic review of ecological factors. Child Psychiatry Hum Dev. (2024) 55:1044–62. 10.1007/s10578-022-01469-7 36422762 PMC9686247

[25] Lenhoff SW Singer J Gottfried M. Thinking Ecologically in Educational Policy and Research. Milton Park: Routledge (2024).

[26] Kim J Jang J Kim B Lee KH. Effect of the PRECEDE-PROCEED model on health programs: a systematic review and meta-analysis. Syst Rev. (2022) 11:213. 10.1186/s13643-022-02092-2 36210473 PMC9549687

[27] Craike M Klepac B Mowle A Riley T. Theory of systems change: an initial, middle-range theory of public health research impact. Res Eval. (2023) 32:603–21. 10.1093/reseval/rvad030

[28] Gentle-Genitty C Taylor J Renguette C. A change in the frame: from absenteeism to attendance. Front Educ. (2020) 4:1–6. 10.3389/feduc.2019.00161

[29] Kearney CA Fensken M Dupont R. The K-16 education movement: common themes across K-12 and higher education systems to inform development and evaluation. Front Educ. (2024) 9:1272297. 10.3389/feduc.2024.1272297

[30] Matranga D Bono F Maniscalco L. Statistical advances in epidemiology and public health. Int J Environ Res Public Health. (2021) 18:3549. 10.3390/ijerph18073549 33805510 PMC8036932

[31] Crear-Perry J Correa-de-Araujo R Lewis Johnson T McLemore MR Neilson E Wallace M. Social and structural determinants of health inequities in maternal health. J Women’s Health. (2021) 30:230–5. 10.1089/jwh.2020.8882 PMC8020519 33181043

[32] Kearney CA Childs J. Translating sophisticated data analytic strategies regarding school attendance and absenteeism into targeted educational policy. Improv Sch. (2023) 26:5–22. 10.1177/13654802231174986

[33] Tsang TK Huang X Guo Y Lau EH Cowling BJ Ip DK. Monitoring school absenteeism for influenza-like illness surveillance: systematic review and meta-analysis. JMIR Public Health Surveill. (2023) 9:e41329. 10.2196/41329 36630159 PMC9878370

[34] Schoeneberger JA. Longitudinal attendance patterns: developing high school dropouts. Clearing House. (2012) 85:7–14. 10.1080/00098655.2011.603766

[35] Robson MG Toscano WA Meng Q Kaden DA. (Eds). Risk Assessment for Environmental Health. Boca Raton, FL: CRC Press (2022).

[36] Leandri M Dalmas L. One health economics: why and how economics should take on the interdisciplinary challenges of a promising public health paradigm. Front Public Health. (2024) 12:1379176. 10.3389/fpubh.2024.1379176 38883196 PMC11177617

[37] Pérez-Marco M Fuster A Gonzálvez C Hamadi SE Havik T. Exploring patterns of school absenteeism: links to school climate in adolescents. Int J Educ Res. (2025) 133:102674. 10.1016/j.ijer.2025.102674

[38] Opara I Thorpe D Lardier DT Jr. School absenteeism and neighborhood deprivation and threat: utilizing the child opportunity index to assess for neighborhood-level disparities in Passaic County, NJ. Urban Educ (Beverly Hills Calif). (2024) 59:2738–66. 10.1177/00420859221125704 39669403 PMC11636610

[39] Stempel H Cox-Martin M Bronsert M Dickinson LM Allison MA. Chronic school absenteeism and the role of adverse childhood experiences. Acad Pediatr. (2017) 17:837–43. 10.1016/j.acap.2017.09.013 28927940

[40] Berman JD McCormack MC Koehler KA Connolly F Clemons-Erby D Davis MF School environmental conditions and links to academic performance and absenteeism in urban, mid-Atlantic public schools. Int J Hyg Environ Health. (2018) 221:800–8. 10.1016/j.ijheh.2018.04.015 29784550 PMC6334301

[41] Kearney CA Ellis K Arcaina VJ. Climate change injustice and school attendance and absenteeism: proximal and distal ecological links. Front Educ. (2024) 9:1455430. 10.3389/feduc.2024.1455430

[42] Schneider MJ. Introduction to Public Health. Burlington, MA: Jones & Bartlett Learning (2020).

[43] Islam KF Awal A Mazumder H Munni UR Majumder K Afroz K Social cognitive theory-based health promotion in primary care practice: a scoping review. Heliyon. (2023) 9:e14889. 10.1016/j.heliyon.2023.e14889 37025832 PMC10070720

[44] Imeri H Toth J Arnold A Barnard M. Use of the transtheoretical model in medication adherence: a systematic review. Res Social Adm Pharm. (2022) 18:2778–85. 10.1016/j.sapharm.2021.07.008 34275751

[45] Askeland KG Bøe T Lundervold AJ Stormark KM Hysing M. The association between symptoms of depression and school absence in a population-based study of late adolescents. Front Psychol. (2020) 11:1268. 10.3389/fpsyg.2020.01268 32655449 PMC7325985

